# Risks of cardiovascular disease and cerebrovascular disease following kidney transplantation: A nationwide, population-based cohort study

**DOI:** 10.7150/ijms.108744

**Published:** 2025-04-13

**Authors:** Tung-Han Tsai, Kuang-Hua Huang, Hsin Chen, Shuo-Yan Gau, Kun-Yu Su, Min-Ling Tsai, Chien-Ying Lee

**Affiliations:** 1 Department of Health Services Administration, China Medical University, Taichung 406040, Taiwan.; 2 School of Medicine, Chung Shan Medical University, Taichung 40201, Taiwan.; 3 Department of Medical Education, Linkou Chang-Gung Memorial Hospital, Taoyuan City 333, Taiwan.; 4 Department of Business Administration, National Taiwan University, Taipei 106, Taiwan.; 5 Department of Pharmacy, Chung Shan Medical University Hospital, Taichung 40201, Taiwan.; 6 Department of Pharmacology, Chung Shan Medical University, Taichung 40201, Taiwan.

**Keywords:** cardiovascular disease, cerebrovascular disease, kidney transplant recipient, cohort study, real-world evidence

## Abstract

**Background:** Kidney transplant recipients (KTRs) have an increased risk for cardiovascular disease (CVD) and cerebrovascular disease (CBD). This study investigated the risks of CVD and CBD following kidney transplantation.

**Materials and methods:** This retrospective cohort study enrolled 3596 KTRs between 2003 and 2017. Propensity Score Matching (PSM) was performed to select patients without a kidney transplant, who were assigned to the control group. Each KTR was matched with five patients without a kidney transplant by sex, age, insured salary, urbanization level, Charlson comorbidity index (CCI), and year of inclusion in the study. A Cox proportional hazards model was employed to investigate the risks of incident CVD and CBD in KTRs after adjusting for relevant variables. Furthermore, we analyzed for CVD and CBD risk 6 months and 1, 3, and 5 years after transplantation.

**Results:** Among KTRs, the CVD incidence rate per 1,000 person-years was 33.98, which was significantly higher than that among patients without a kidney transplant. After adjusting for confounding variables, KTRs had a significantly higher risk of CVD (adjusted hazard ratio [aHR], 1.74; 95% confidence interval [CI], 1.58-1.93) than did patients without a kidney transplant. Regarding cumulative incidence, the risk of CVD increased over time. Among the four follow-up periods we assessed, the 5-year follow-up period had the highest CVD risk (aHR, 1.35; 95% CI, 1.17-1.56), followed by the 3-year follow-up period (aHR, 1.34; 95% CI, 1.13-1.59). KTRs also had a significantly higher risk of CBD (aHR, 1.43; 95% CI, 1.23-1.68) than did patients without a kidney transplant.

**Conclusion:** CVD risk is higher among KTRs than among those without a kidney transplant, and this risk increases over time. CBD risk was also higher among KTRs. Large, randomized controlled prospective studies are needed to thoroughly evaluate the relationship between kidney transplantation and the risks of CVD and CBD.

## Introduction

Kidney transplantation is the most effective treatment for patients with irreversible chronic kidney disease 1. Kidney transplantation yields better outcomes in terms of quality of life and mortality risk compared with dialysis 2. The most common cause of graft failure is death with a functioning graft. Cardiovascular disease (CVD), infections, and cancer are the leading causes of death in kidney transplant recipients (KTRs) 3.

Observational studies and substantial evidence show that CVD is more prevalent among KTRs than in the general population 4, 5, 6. Dialysis poses a greater risk of cardiovascular mortality than does kidney transplantation 7, 8. CVD is the leading cause of morbidity and mortality among KTRs and the primary reason for posttransplant hospitalization 9. CVD poses a threat to the long-term survival of KTRs 10. New-onset cerebrovascular disease (CBD) is relatively common among KTRs 11-15. All forms of CBD are linked to increased mortality risk 14. Clinicians must understand the risks of CVD and CBD following kidney transplantation to prevent complications. Accordingly, this retrospective cohort study investigated the risks of CVD and CBD following kidney transplantation between 2003 and 2017. Data were obtained from Taiwan's National Health Insurance Research Database (NHIRD).

## Materials and methods

### Data sources

This study conducted a secondary analysis of the 2002-2022 NHIRD. The NHIRD is managed by Taiwan's Ministry of Health and Welfare. The NHIRD contains data for all beneficiaries covered by the National Health Insurance program, a government-run, single-payer National Health Insurance program established in 1995. This insurance program covers approximately 99% of Taiwan's population. Disease diagnoses within the database are coded using the *International Classification of Diseases, Ninth Revision, Clinical Modification* (*ICD-9-CM*) and *International Classification of Diseases, Tenth Revision, Clinical Modification* (*ICD-10-CM*) systems. Before 2015, diagnoses were reported using ICD-9-CM, while from 2016 onward, ICD-10-CM has been used. The NHIRD is commonly employed to inform clinical decision-making and health-care policy development 16, 17.

### Ethics approval

Data in the NHIRD are anonymized; therefore, this study was exempt from the need to obtain informed consent. The study protocol received ethical approval from the Institutional Review Board of Chung Shan Medical University Hospital, Taiwan (No. CSMUH CS2-21134).

### Study participants

**Figure 1:**
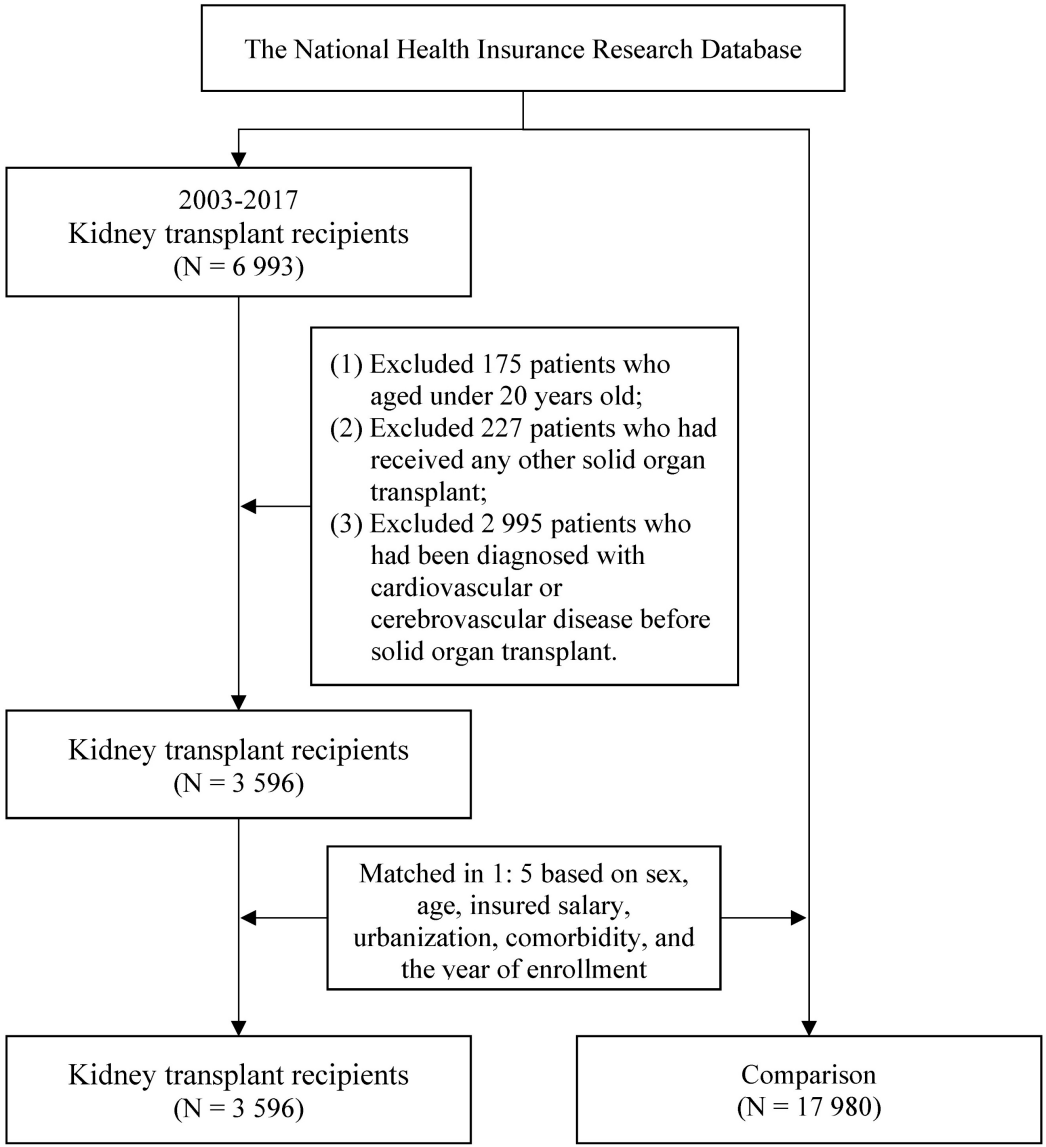
Flowchart of the patient selection process

The study enrolled patients aged older than 20 years who received a kidney transplant between 2003 and 2017. Kidney transplantation was identified using *ICD-9-CM code* V42.0 and *ICD-10-CM code* Z94.0. In Taiwan, organ transplantation is classified as a major catastrophic illness under the National Health Insurance (NHI) program. Patients who have successfully undergone organ transplantation receive a catastrophic illness certificate. Considering potential factors such as surgical failure or overseas transplantation, this study identified kidney transplant recipients using diagnosis codes from the catastrophic illness registry instead of procedure codes. The index date for kidney transplant patients was defined based on the 'effective start date' recorded in the registry, marking the beginning of the observation period. Patients were excluded if they had received any other solid organ transplant or had a CVD or CBD before receiving a kidney transplant. The control group consisted of individuals who had not undergone organ transplantation during the study period. To reduce potential confounding caused by unbalanced covariates in non-experimental settings, we employed 1:5 propensity score matching for each KTR based on the year of enrollment (i.e., 2003, 2004, 2005, until 2017) to establish a matched cohort. To minimize bias, only individuals without a prior diagnosis of cardiovascular disease (CVD) or cerebrovascular disease (CBD) before the enrollment year were eligible for inclusion in the control group. A propensity score is a probability that is calculated using a logistic regression model. The score is a unit of a certain characteristic assigned to a patient who received a kidney transplant. These scores can help reduce or eliminate selection bias in observational studies by accounting for the characteristics of control individuals. Sex, age, insured salary, urbanization level, and Charlson comorbidity index (CCI) were selected as characteristics for matching. Specifically, an optimized matching approach was used with a caliper width of 0.2 of the pooled standard deviation of the logit of the propensity score. If a control subject had experienced the outcome event prior to matching, they were completely excluded from the analysis. Additionally, subjects that could not be successfully matched were also excluded to ensure the integrity of the comparison. This approach ensured comparability between groups and improved the validity of our findings. After matching, the study comprised 3596 KTRs (KTR group) and 17 980 patients without a kidney transplant (control group). The patient selection process is presented in **Figure 1**.

### Study design

This retrospective cohort study investigated the risk of CVD and CBD by using data from Taiwan's NHIRD. The CVDs investigated comprised acute myocardial infarction (*ICD-9-CM* 410; *ICD-10-CM* I21-I22), atrial fibrillation (*ICD-9-CM* 427.31; *ICD-10-CM* I48.0, II48.1, I48.2, I48.91), ischemic heart disease (*ICD-9-CM* 410-414; *ICD-10-CM* I20-I25), heart failure (*ICD-9-CM* 428; *ICD-10-CM* I50), and peripheral artery disease (*ICD-9-CM* 443.9; *ICD-10-CM* I73.9). CBD comprised ischemic stroke (*ICD-9-CM* 443-435, 437; *ICD-10-CM* G45, G46, I63, I65-I67, I69) and hemorrhagic stroke (*ICD-9-CM* 430-432; *ICD-10-CM* I60-I62). The definitions of CVD and CBD were based on diagnostic criteria requiring at least three occurrences as a principal diagnosis in outpatient records or at least one occurrence as any listed diagnosis during hospitalization within the follow-up period. The incident date of CVD or CBD was defined as the date of the first recorded diagnosis. The approval date of KTR was defined as the start of the observation period. For the matched comparison group, the same date was assigned as the start of the observation period. All patients were followed up from the observation start date until death, diagnosis of CVD, CBD, or the end date of 2022.

The control variables included sex, age, insured salary, urbanization level, CCI, and related comorbidities. Age was categorized as ≤40, 41-50, 51-60, and ≥61 years. Insured salary was categorized into four groups: ≤ 19,200, 19,201-22,800, 22,801-42,000, and ≥ 42,001 New Taiwan Dollars (NTD). For urbanization level, we classified the insured regions of study subjects into seven levels based on previous research definitions, with level 1 being the highest and level 7 the lowest 18. CCI was categorized as 0, 1, 2, and ≥ 3. The comorbidities included in our analysis were hypertension (*ICD-9-CM* 401-405; *ICD-10-CM* I10-I13, I15), hyperlipidemia (*ICD-9-CM* 272; *ICD-10-CM* E78), diabetes mellitus (*ICD-9-CM* 250; *ICD-10-CM* E10-E14), chronic kidney disease (*ICD-9-CM* 585; *ICD-10-CM* N18), hyperuricemia (*ICD-9-CM* 790.6; *ICD-10-CM* E79.0), anxiety (*ICD-9-CM* 300.0; *ICD-10-CM* F40-F41), depression (*ICD-9-CM* 296.2-296.3; *ICD-10-CM* F32-F33), and sleep disturbance (*ICD-9-CM* 780; *ICD-10-CM* G47.9).

### Statistical analysis

All statistical analyses were conducted using SAS software version 9.4 (SAS Institute, Cary, NC, USA). Statistical significance was indicated by *p* < .05. Chi-square tests were used to compare the distributions of baseline characteristics between KTRs and patients without a kidney transplant. Since the number and proportion of deaths during the follow-up period were lower than those of CVD events, we treated death as a censoring event rather than a competing event. A Cox proportional hazards model was employed to investigate the risk of incident CVD and CBD in KTRs after adjustment for all relevant variables. Results are presented as hazard ratios (HRs) with 95% confidence intervals (CIs). The risks of incident CVD and CBD following kidney transplantation may change over time; therefore, we conducted a sensitivity analysis to estimate the risks of CVD and CBD over several follow-up periods, namely 6 months and 1, 3, and 5 years.

## Results

The baseline characteristics of the participants are given in **Table 1**. In total, 49.25% of the KTRs were men, and the mean age of KTRs was 45.69 ± 11.30 years. Furthermore, 32.81%, 31.65%, 26.28%, and 9.26% of KTRs were aged ≤40, 41-50, 51-60, and ≥61 years, respectively. After propensity score matching, no significant differences in sex, age, insured salary, urbanization, and CCI between the groups were observed (*p* > 0.05). Among KTRs, 54.06% had hypertension, 6.03% had hyperlipidemia, 14.40% had diabetes mellitus, 66.60% had chronic kidney disease, and 1.06% had depression. The distribution of the aforementioned comorbidities in the KTR group significantly differed from that in the control group (*p* < .001).

CVD incidence rates are shown in **Table 2**. The average follow-up duration was 10.02 ± 5.58 years. In total, 4424 patients (20.50%) developed CVD, and the CVD incidence rate per 1,000 person-years was 19.58. Among KTRs, the CVD incidence rate per 1,000 person-years was 33.98, which was significantly higher than that in the control group (*p* < .001). CVD incidence increased with age. Hypertension, hyperlipidemia, diabetes mellitus, or chronic kidney disease increased the risk of CVD.

Findings on CVD risk are shown in** Table 3**. After confounding variables were adjusted for, the risk of CVD was found to be significantly higher in the KTR group (adjusted HR [aHR], 1.74; 95% CI, 1.58-1.93) than in the control group. The risk of CVD was higher among men (aHR, 1.18; 95% CI, 1.12-1.26) than among women. The risk of CVD was increased by the following comorbidities: hypertension (aHR, 1.18; 95% CI, 1.10-1.26), hyperuricemia (aHR, 1.19; 95% CI, 1.07-1.33), diabetes mellitus (aHR, 1.28; 95% CI, 1.19-1.38), and chronic kidney disease (aHR, 1.16; 95% CI, 1.04-1.29).

Findings on CVD risk stratified by follow-up duration are shown in **Table 4**. The KTR group had a higher risk of CVD than did the control group during 3-year (aHR, 1.34; 95% CI, 1.13-1.59) and 5-year (aHR, 1.35; 95% CI, 1.17-1.56) follow-up windows.

CBD risk is shown in **Table 5**. After confounding variables were adjusted for, the risk of CBD was found to be significantly higher in the KTR group (aHR, 1.43; 95% CI, 1.23-1.68) than in the control group. The KTR group had a lower risk of CBD than did the control group within a 6-month follow-up window (aHR, 0.53; 95% CI, 0.28-0.99) and a higher risk of CBD in a 5-year follow-up window (aHR, 1.26; 95% CI, 1.01-1.57).

## Discussion

This large-scale retrospective observational study provides real-world findings regarding the risk of CVD following kidney transplantation. The study demonstrated that CVD risk was higher in the KTR group than in the control group and that KTRs with comorbid hypertension, hyperlipidemia, diabetes mellitus, chronic kidney disease, and depression were at further risk. Male sex and older age were risk factors for CVD, and this risk increased over time following transplantation. CBD risk was also higher in the KTR group than in the control group.

The risks of CVD and mortality are lower among KTRs than among patients receiving dialysis. Nevertheless, these risks are higher among KTRs than in the general population 19. KTRs have double the risk of cardiovascular mortality compared with the general population 20, 21. The high incidence of CVD after kidney transplantation is primarily due to the presence and accumulation of traditional risk factors both before and after the transplant 22.

Male and older-adult KTRs, especially those aged 51-60 years, have a higher risk of CVD. The incidence of CV remains high after transplantation. A study using survival model analysis identified the following as key predictors of cardiovascular events in KTRs: male sex, older age, previous CVD, pretransplant smoking, and posttransplant diabetes mellitus 23. These factors increase the risk of cardiovascular events 23. Age and sex are risk factors for vascular disease 24. CVD accounts for one-third of all deaths following transplantation, with individuals aged 50 years or older facing a significantly higher risk than the general population 9.

KTRs with comorbid hypertension, hyperlipidemia, diabetes mellitus, chronic kidney disease, and depression had a higher risk of CVD than did KTRs without these comorbidities. Traditional cardiovascular risk factors, such as hyperlipidemia, hypertension, and diabetes mellitus, are common in KTRs, partly due to the effects of immunosuppressive drugs. These factors contribute to adverse outcomes and increase the burden of CVD after transplantation 14, 25. Hypertension is a major risk factor for atherosclerotic CVD, which is the leading cause of premature death and a major factor in graft failure among transplant recipients 26. Although hypertension is a significant risk factor for renal graft survival, controlling hypertension alone does not seem to improve outcomes. Graft survival is more affected by the severity of graft dysfunction at the start of observation, regardless of blood pressure control 27. Dyslipidemia is common among KTRs, even when renal function remains normal or nearly normal 28. Dyslipidemia is an important risk factor for CVD 29. The prevalence of hyperlipidemia among KTRs has been estimated to reach up to 80% 30. Abnormal glucose metabolism significantly increases the risk of fatal and nonfatal cardiovascular events 31, with a threefold higher risk of cardiac death or nonfatal acute myocardial infarction compared with patients without diabetes 32. Patients with pretransplant diabetes mellitus (PreDM) had an increased risk of mortality from cardiovascular diseases compared to those without diabetes 33, 34. Another study also indicted that PreDM patients have the highest incidence of major adverse cardiovascular events and cardiac-related mortality following transplantation 35. Patients with PreDM experienced significantly lower survival rates, with up to a 50% higher risk of death while on the transplant waiting list and a 70% increased risk of CVD and all-cause mortality 36, 37. Chronic kidney disease increases the risk of death among KTRs, and KTRs with chronic kidney disease are more likely to require dialysis than are KTRs without chronic kidney disease 38. CVD is a significant cause of morbidity and mortality among patients with chronic kidney disease 21. Progressive renal dysfunction increases the risk of cardiovascular complications following transplantation 4. Chronic kidney disease increases the risk of adverse outcomes, with CVD being the leading cause of death in this population 39. Cardiovascular mortality increases as kidney function declines, even after adjusting for traditional risk factors, indicating that chronic kidney disease is an independent risk factor for CVD 40. In advanced chronic kidney disease stages (3b-4), 40%-45% of patients die from CVD rather than progressing to end-stage kidney disease requiring transplantation or dialysis 41. Depression and anxiety are consistently identified as the most prevalent psychiatric disorders among organ donors following donation 42. Anxiety and depression are associated with higher morbidity following kidney transplantation 43. A meta-analysis associated depression but not anxiety with an increased risk of posttransplant mortality 44. Despite progress in renal transplantation, depression is still a common and problematic comorbidity that remains largely overlooked. In this context, depression is associated with poorer outcomes, including reduced graft survival 45.

CVD risk increased over time following kidney transplantation. Among the four follow-up periods we assessed, the 5-year follow-up period had the highest CVD risk, followed by the 3-year follow-up period. CVD has become a major contributor to graft loss, especially after the first year following transplantation 4. We applied competing risk methodology and found that the cumulative incidence of cardiovascular events was 5.0% at 1 year after transplantation, 8.1% at 5 years, and 11.9% at 10 years 23. The cumulative incidence of posttransplant myocardial infarction was 4.3% following transplantation, 4.1%-4.5% at 6 months, 5.6% at 12 months, and 11.1% at 36 months 46. The cumulative incidence of coronary heart disease following transplantation was 3.1% at 1 year, 5.2% at 3 years, and 7.6% at 5 years 47.

Our study indicates that the risk of CBD was higher in KTRs compared to non-transplant patients during the 5-year follow-up period. In contrast, the 6-month follow-up period was associated with a lower CBD risk (aHR 0.53, 95% CI: 0.28-0.99). However, the lower limit of the confidence interval is close to negligible. The incidence of posttransplantation stroke in KTRs varies from 1.1% to 8.0% 11-15. Single-center studies have demonstrated the adverse mortality implications of posttransplantation cerebrovascular disease events in KTRs 13, 48. The cumulative 3-year incidence of new-onset cerebrovascular events after kidney transplantation was 6.8%. Among patients on the waiting list, this incidence was 11.8%, and among those with graft loss, this was 11.2% 14. Transplantation reduced the risk of cerebrovascular events by 34% compared with remaining on the waiting list, and the risk of cerebrovascular events increased by more than 150% after graft failure 14. Cerebrovascular events are associated with negative implications for mortality 14. Kidney function within the first year after transplantation has consistently been recognized as an important factor that affects longer-term graft survival 49, 50. KTRs experience a gradual decline in glomerular filtration rate over time, which is strongly associated with donor age and aortic stiffness after the first year following transplantation 51. A study has shown evidence of the involvement of kidney disease in both the early and later phases of cerebrovascular atherosclerosis and cerebral small vessel disease 52. Kidney dysfunction was linked to an increased risk of stroke, regardless of the etiological subtype 53. The incidence of stroke in KTRs was 5% in the first year following transplantation and 9.4% in the second year 15. This study revealed that the incidence of stroke was relatively lower in the early stages after transplantation but increased in the later stages. Our research shows that the risk of CBD was higher in KTRs than in non-transplant patients over the 5-year follow-up period. On the other hand, the 6-month follow-up period was linked to a lower risk of CBD. This may be associated with initial improvement in kidney function in the early post-transplant period, followed by a gradual decline over time. However, further well-designed controlled prospective studies are needed to confirm whether CBD risk continues to increase over time following kidney transplantation.

CVD risk was higher in the KTR group than in the control group, and this risk increased over time. CBD risk was also higher in the KTR group. Clinicians must comprehensively understand the risks of CVD and CBD following kidney transplantation to minimize complications.

The present study has several strengths. First, it used a nationwide, population-based sample, selecting patients from the entire Taiwanese population and following them over a long period. This approach provided a large, representative sample with high statistical value and minimized selection bias. Second, we investigated the risks of CVD and CBD in KTRs at different time points after transplantation (i.e., 6 months and 1, 3, and 5 years).

This study has some limitations. First, selective reporting bias and data errors may have resulted from the study's retrospective cohort design. Second, the study used the NHIRD, which is a secondary database. Because the NHIRD lacks laboratory details and medical examination data, we were unable to evaluate diagnostic criteria. Third, the NHIRD lacks data on smoking habits, alcohol consumption, body mass index, personal medical history, physical activity, and dietary habits, all of which are linked to CVD and CBD risk. Fourth, all diagnoses in this study were based on *ICD-9-CM* or *ICD-10-CM* classifications. Taiwan's Bureau of National Health Insurance enhances diagnostic accuracy by randomly reviewing charts and conducting patient interviews, ensuring that analyses using NHIRD data are valid and accurate. Nonetheless, we acknowledge that some confounding variables may not have been controlled for in this study. Last, some comorbidities, such as metabolic syndrome, obesity, and obstructive sleep apnea, are associated with the occurrence of CVD and CBD. However, due to the limited number of patients with these conditions, their inclusion could lead to overdispersion in the regression analyses. Therefore, they were not incorporated into this study. This study focused on the incidence of CVD and CBD after kidney transplantation, without further analysis of the underlying reasons for transplantation. While end-stage renal disease (ESRD) is the most common indication, other conditions, such as congenital anomalies, genetic disorders, or rare kidney diseases, may also lead to kidney transplantation. Different transplantation indications could potentially influence post-transplant CVD and CBD risk. Future studies should consider incorporating the causes of transplantation to better understand their impact on cardiovascular and cerebrovascular outcomes.

## Figures and Tables

**Table 1:** Distribution of baseline characteristics of kidney transplant recipients and the comparison.

Variables	Total		Comparison		Kidney transplant recipients		p-value
	N	%	N	%	N	%	
Total	21,576	100.00	17,980	100.00	3,596	100.00	
Sex ^1^							0.995
Female	10,951	50.76	9,126	50.76	1,825	50.75	
Male	10,625	49.24	8,854	49.24	1,771	49.25	
Age (year) ^1^							1.000
≤40	7,096	32.89	5,916	32.90	1,180	32.81	
41-50	6,816	31.59	5,678	31.58	1,138	31.65	
51-60	5,671	26.28	4,726	26.28	945	26.28	
≥61	1,993	9.24	1,660	9.23	333	9.26	
Mean ± SD	45.36 ± 14.27		45.30 ± 14.80		45.69 ± 11.30		
Insured salary (NTD.) ^1^							0.999
≤19,200	7,105	32.93	5,922	32.94	1,183	32.90	
19,201-22,800	4,435	20.56	3,694	20.55	741	20.61	
22,801-42,000	5,439	25.21	4,535	25.22	904	25.14	
≥42,001	4,597	21.31	3,829	21.30	768	21.36	
Urbanization ^1^							1.000
Level 1	7,111	32.96	5,921	32.93	1,190	33.09	
Level 2	7,313	33.89	6,095	33.90	1,218	33.87	
Level 3	3,279	15.20	2,731	15.19	548	15.24	
Level 4	2,548	11.81	2,125	11.82	423	11.76	
Level 5	303	1.40	253	1.41	50	1.39	
Level 6	480	2.22	402	2.24	78	2.17	
Level 7	542	2.51	453	2.52	89	2.47	
CCI score ^1^							1.000
0	2,083	9.65	1,735	9.65	348	9.68	
1	875	4.06	730	4.06	145	4.03	
2	9,189	42.59	7,657	42.59	1,532	42.60	
≥3	9,429	43.70	7,858	43.70	1,571	43.69	
Enrolled year ^1^							1.000
2003	2,376	11.01	1,980	11.01	396	11.01	
2004	2,064	9.57	1,720	9.57	344	9.57	
2005	1,938	8.98	1,615	8.98	323	8.98	
2006	1,800	8.34	1,500	8.34	300	8.34	
2007	1,530	7.09	1,275	7.09	255	7.09	
2008	1,458	6.76	1,215	6.76	243	6.76	
2009	1,320	6.12	1,100	6.12	220	6.12	
2010	1,290	5.98	1,075	5.98	215	5.98	
2011	1,260	5.84	1,050	5.84	210	5.84	
2012	1,116	5.17	930	5.17	186	5.17	
2013	1,026	4.76	855	4.76	171	4.76	
2014	1,140	5.28	950	5.28	190	5.28	
2015	1,140	5.28	950	5.28	190	5.28	
2016	978	4.53	815	4.53	163	4.53	
2017	1,140	5.28	950	5.28	190	5.28	
Comorbidities							
Hypertension							<0.001
No	16,346	75.76	14,694	81.72	1,652	45.94	
Yes	5,230	24.24	3,286	18.28	1,944	54.06	
Hyperlipidemia							0.005
No	20,037	92.87	16,658	92.65	3,379	93.97	
Yes	1,539	7.13	1,322	7.35	217	6.03	
Diabetes mellitus							<0.001
No	17,081	79.17	14,003	77.88	3,078	85.60	
Yes	4,495	20.83	3,977	22.12	518	14.40	
Chronic kidney disease							<0.001
No	18,308	84.85	17,107	95.14	1,201	33.40	
Yes	3,268	15.15	873	4.86	2,395	66.60	
Hyperuricemia							<0.001
No	21,399	99.18	17,898	99.54	3,501	97.36	
Yes	177	0.82	82	0.46	95	2.64	
Anxiety							<0.001
No	20,787	96.34	17,251	95.95	3,536	98.33	
Yes	789	3.66	729	4.05	60	1.67	
Depression							<0.001
No	21,148	98.02	17,590	97.83	3,558	98.94	
Yes	428	1.98	390	2.17	38	1.06	
Sleep disturbance							0.002
No	18,327	84.94	15,334	85.28	2,993	83.23	
Yes	3,249	15.06	2,646	14.72	603	16.77	

^1^ Variables for propensity score matching.

**Table 2:** Incidence rate of cardiovascular disease.

Variables	Cardiovascular disease			
	Events	%	IR ^1^	p-value
Total	4,424	20.50	19.58	
Patients				<0.001
Comparison	3,292	18.31	17.09	
Kidney transplant recipients	1,132	31.48	33.98	
Sex				0.007
Female	2,166	19.78	18.19	
Male	2,258	21.25	21.12	
Age (year)				<0.001
≤40	1,026	14.46	12.31	
41-50	1,637	24.02	22.28	
51-60	1,388	24.48	25.56	
≥61	373	18.72	25.09	
Insured salary (NTD.)				<0.001
≤19,200	1,594	22.43	20.29	
19,201-22,800	772	17.41	18.05	
22,801-42,000	1,132	20.81	19.55	
≥42,001	926	20.14	19.80	
Urbanization				0.036
Level 1	1,499	21.08	19.93	
Level 2	1,535	20.99	19.95	
Level 3	605	18.45	18.01	
Level 4	505	19.82	19.00	
Level 5	59	19.47	19.10	
Level 6	98	20.42	20.67	
Level 7	123	22.69	21.07	
CCI score				<0.001
0	339	16.27	12.70	
1	185	21.14	17.60	
2	2,003	21.80	20.22	
≥3	1,897	20.12	21.15	
Comorbidities				
Hypertension				<0.001
No	3,038	18.59	17.01	
Yes	1,386	26.50	29.25	
Hyperlipidemia				<0.001
No	4,031	20.12	19.10	
Yes	393	25.54	26.42	
Diabetes mellitus				<0.001
No	3,321	19.44	17.98	
Yes	1,103	24.54	27	
Chronic kidney disease				<0.001
No	3,463	18.92	17.54	
Yes	961	29.41	33.63	
Hyperuricemia				<0.001
No	4,367	20.41	19.46	
Yes	57	32.20	36.23	
Anxiety				0.803
No	4,265	20.52	19.57	
Yes	159	20.15	19.70	
Depression				0.216
No	4,326	20.46	19.49	
Yes	98	22.90	24.78	
Sleep disturbance				0.002
No	3,692	20.15	19.11	
Yes	732	22.53	22.34	

^1^ Incidence rate per 1,000 person-year.

**Table 3:** Risk estimated for incident cardiovascular disease using the Cox proportional hazards model.

Variables	Cardiovascular disease				
	Adjusted HR	95% CI			p-value
Patients					
Comparison (ref.)	1				
Kidney transplant recipients	1.74	1.58	-	1.93	<0.001
Sex					
Female (ref.)	1				
Male	1.18	1.12	-	1.26	<0.001
Age (year)					
≤40 (ref.)	1				
41-50	1.78	1.64	-	1.92	<0.001
51-60	1.97	1.81	-	2.14	<0.001
≥61	1.82	1.61	-	2.05	<0.001
Insured salary (NTD)					
≤19,200 (ref.)	1				
19,201-22,800	0.86	0.79	-	0.94	0.001
22,801-42,000	0.93	0.87	-	1.01	0.084
≥42,001	0.86	0.79	-	0.93	0.000
Urbanization					
Level 1 (ref.)	1				
Level 2	0.98	0.91	-	1.05	0.560
Level 3	0.89	0.81	-	0.98	0.021
Level 4	0.96	0.87	-	1.07	0.486
Level 5	0.92	0.71	-	1.19	0.519
Level 6	0.94	0.77	-	1.16	0.569
Level 7	1.06	0.88	-	1.27	0.559
CCI score					
0 (ref.)	1				
1	1.24	1.04	-	1.49	0.019
2	1.44	1.28	-	1.63	<0.001
≥3	1.35	1.20	-	1.53	<0.001
Comorbidities (Yes vs No [ref.])					
Hypertension	1.18	1.10	-	1.26	<0.001
Hyperlipidemia	1.19	1.07	-	1.33	0.001
Diabetes mellitus	1.28	1.19	-	1.38	<0.001
Chronic kidney disease	1.16	1.04	-	1.29	0.007
Hyperuricemia	1.25	0.96	-	1.63	0.098
Anxiety	0.98	0.84	-	1.15	0.820
Depression	1.25	1.02	-	1.53	0.030
Sleep disturbance	1.06	0.98	-	1.15	0.145

**Table 4:** Analysis for cardiovascular disease risk at different follow-up times.

Variables	Cardiovascular disease						
	Events	%	Adjusted HR	95% CI			p-value
In 6 months							
Patients							
Comparison (ref.)	300	1.67	1				
Kidney transplant recipients	79	2.20	1.23	0.86	-	1.75	0.253
In 1 year							
Patients							
Comparison (ref.)	512	2.85	1				
Kidney transplant recipients	138	3.84	1.10	0.84	-	1.44	0.479
In 3 years							
Patients							
Comparison (ref.)	1,081	6.01	1				
Kidney transplant recipients	355	9.87	1.34	1.13	-	1.59	<0.001
In 5 years							
Patients							
Comparison (ref.)	1,589	8.84	1				
Kidney transplant recipients	506	14.07	1.35	1.17	-	1.56	<0.001

**Table 5:** Risk estimated for incident cerebrovascular disease using the Cox proportional hazards model.

Variables	Cerebrovascular Disease				
	Adjusted HR	95% CI			p-value
Patients					
Comparison (ref.)	1				
Kidney transplant recipients	1.43	1.23	-	1.68	<0.001
At different follow-up time					
In 6 months					
Comparison (ref.)	1				
Kidney transplant recipients	0.53	0.28	-	0.99	0.049
In 1 year					
Comparison (ref.)	1				
Kidney transplant recipients	0.82	0.52	-	1.30	0.394
In 3 years					
Comparison (ref.)	1				
Kidney transplant recipients	1.07	0.81	-	1.41	0.658
In 5 years					
Comparison (ref.)	1				
Kidney transplant recipients	1.26	1.01	-	1.57	0.048
